# Association of serum adipocytokine levels with cardiac autonomic neuropathy in type 2 diabetic patients

**DOI:** 10.1186/1475-2840-11-24

**Published:** 2012-03-13

**Authors:** Chan-Hee Jung, Bo-Yeon Kim, Chul-Hee Kim, Sung-Koo Kang, Sang-Hee Jung, Ji-Oh Mok

**Affiliations:** 1 Division of Endocrinology and Metabolism, Department of Internal Medicine, Soonchunhyang University School of Medicine, #108 Jung-Dong, Wonmi-Ku, Bucheon, 110-746 Kyunggi-Do, Korea; 2 Department of Obstetrics and Gynecology, Cha University School of Medicine, Bundang, Korea

**Keywords:** Cardiac autonomic neuropathy, heart rate variability, leptin, TNF- alpha, adiponectin, type 2 diabetes mellitus

## Abstract

**Background:**

Cardiac autonomic neuropathy (CAN) is a common complication of diabetes associated with poor prognosis. In addition, the autonomic imbalance is associated with cardiovascular disease (CVD) in diabetes. It is thought that adipocytokines contribute to the increased risk of vascular complications in patients with type 2 diabetes mellitus (T2DM). However, literature data on the association between CAN with adipocytokines such as leptin, tumor necrosis factor-alpha (TNF-alpha), adiponectin in subjects with T2DM is limited.

Therefore, in the present study, we examined the relationship between fasting serum leptin, TNF- alpha and adiponectin and CAN in Korean T2DM patients.

**Methods:**

A total of 142 T2DM patients (94 males, 48 females) were recruited. CAN was assessed by the five tests according to the Ewing's protocol and the time and frequency domain of the heart rate variability (HRV) was evaluated. Serum TNF-alpha and adiponectin levels were measured using enzyme-linked immunosorbent assay and serum leptin levels were measured using radioimmunoassay.

**Results:**

Although, the mean levels of leptin, TNF-alpha and adiponectin were not significantly different between the groups with and without CAN, the levels of leptin and adiponectin had a tendency to increase as the score of CAN increased (p = 0.05, p = 0.036). Serum leptin levels demonstrated a negative correlation with low frequency (LF) in the upright position (p = 0.037). Regarding TNF-alpha, a significant negative correlation was observed with SDNN and RMSSD in the upright position (p = 0.023, p = 0.019). Adiponectin levels were not related to any HRV parameters. Multivariate logistic regression analysis demonstrated that the odds of CAN increased with a longer duration of diabetes (1.25, [1.07-1.47]) and higher homeostatic model of assessment-insulin resistance (HOMA-IR) (5.47, [1.8-16.5]). The relative risks for the presence of CAN were 14.1 and 51.6 for the adiponectin 2^nd^, 3^rd ^tertiles when compared with first tertile (p-value for trend = 0.022).

**Conclusions:**

In the present study, the higher serum adiponectin levels and HOMA-IR were associated with an increased risk for the presence of CAN. Also, the CAN score correlated with the serum adiponectin. Serum adipocytokines such as leptin and TNF-alpha were significantly correlated with parameters of HRV, representative markers of CAN. Future prospective studies with larger number of patients are required to establish a direct relationship between plasma adipocytokine concentrations and the development or severity of CAN.

## Background

Although cardiac autonomic neuropathy (CAN) is one of the most common complications of diabetes, it is commonly overlooked. CAN represents a significant cause of morbidity and mortality in diabetic patients and is associated with a high risk of cardiac arrhythmias and sudden death, possibly related to silent myocardial ischemia [1-3].

Autonomic imbalance, characterized by a hyperactive sympathetic system and a hypoactive parasympathetic system, is associated with cardiovascular disease (CVD) in diabetes [4,5]. The heart rate variability (HRV) has been used to assess autonomic imbalances, diseases and mortality. Low HRV and sympathetic overactivity are predictors of CVD.

Although hyperglycemia plays a key role in the development of CAN, strict glycemic control cannot abolish the risk of CAN, suggesting the involvement of other contributing factors to its development and the need for their identification [6].

Adipose tissue secretes adipocytokines which have an impact on glucose and lipid metabolism, the inflammatory process and other bioactivities [7,8]. It is thought that adipocytokines contribute to the increased risk of vascular complications in patients with type 2 diabetes mellitus (T2DM) by modulating vascular function and affecting inflammatory processes [7,9]. In addition, the role of the adipocytokines on HRV in various conditions has attracted considerable attention.

Leptin may play a role as a potential marker for the diagnosis of obesity-related disease and has been shown to stimulate the sympathetic nervous system (SNS) [10,11]. In addition, leptin exerts an atherogenic and angiogenetic effect and is associated with the development of T2DM and CVD [12]. However, studies examining the impact of leptin on CAN or HRV in T2DM are limited.

There is now convincing data demonstrating that diabetes includes an inflammatory component thought to be related to diabetic complications. Several reports support the hypothesis that dysregulation of the TNF superfamily may be involved in the development of diabetic vascular complications [13]. However, literature data regarding the association between CAN and TNF-alpha (TNF-α) in subjects with T2DM is limited.

Adiponectin, the most abundant adipocytokine, was found to be decreased in conditions such as obesity, insulin resistance, T2DM, its macrovascular complications and coronary artery disease (CAD) [14]. Siitonen et al. reported that single nucleotide polymorphisms in adiponectin receptors may modify the risk of CVD in individuals with impaired glucose tolerance [15]. In addition, hypoadiponectinemia is assciated with SNS overactivity [16]. However, there is still limited information on regarding the relationship between plasma adiponectin, T2DM and cardiac autonomic nervous function.

To our knowledge, none of the previously published studies investigated the effects of leptin, TNF-α, and adiponectin on the presence of CAN in T2DM. Therefore, in the present study, we examined the relationship between fasting serum leptin, TNF-α, adiponectin and CAN in 142 Korean T2DM patients.

## Methods

### Patients

We recruited 170 diabetic patients who underwent cardiac autonomic function tests at diabetes clinic of Soonchunhyang University Bucheon Hospital, from January 2009 to May 2011. Among the total 170 patients, those with type 1 diabetes, and were not available of fasting serum samples were excluded. Also, six patients taking beta-blockers were excluded. Finally, this study was performed on 142 T2DM patients (94 males and 48 females, mean age: 52.4 years). We reviewed detailed demographic data, biochemical data, clinical and treatment history using medical records. All patients were informed of the purpose of the study and their consent was obtained. The study was approved by the Institutional Review Board of Soonchunhyang University School of Medicine, Bucheon Hospital.

### Cardiac autonomic function test

Autonomic function tests (AFT) were performed in the morning at a quiet room by the same operator and were analyzed by one investigator. Subjects were advised to refrain from smoking, eating, and coffee consumption for at least 2 hour before the tests.

CAN was assessed by the five standard cardiovascular reflex tests according to the Ewing's protocol [17]. Three of these measurements mainly assess parasympathetic function; heart rate responses to deep breathing (beat-to-beat variation), to standing (30:15 ratio), and to the Valsalva maneuver. The other two tests mainly assess sympathetic function; blood pressure responses to standing and a sustained handgrip. The heart rate response to deep breathing, standing, and the Valsalva maneuver were assessed automatically from ECG recordings using the DICAN evaluation system (Medicore Co. Ltd, Korea). The results of each of the above five tests for the detection of CAN were classified into three categories based on the severity of abnormality detected, and each of them was given a definite point as described by Bellavere et al. [18].

#### Determination of the CAN score

The severity of CAN was quantitated by summation of points obtained from each of the five tests, where each test was given a point of 0, 0.5, or 1 if it yielded normal, borderline, or abnormal values, respectively. Consequently, the minimum and maximum autonomic neuropathy points were 0 and 5, respectively. CAN was defined as the presence of at least two abnormal tests or an autonomic neuropathy points of ≥ 2 [19].

The CAN score was categorized as follows: CAN score 0 (total points 0), CAN score 1 (points 0.5-1.5), CAN score 2 (points 2-3), and CAN score 3 (points ≥ 3.5). CAN was considered absent, early, definite, or severe if the CAN scores were 0, 1, 2, or 3, respectively.

#### Time domain and frequency domain of heart rate variability

##### Time domain variables

The standard deviation of normal-to-normal RR intervals (SDNN; ms, correlated to total autonomic activity) and the square root of the mean of the squares of differences between successive RR intervals (RMSSD; ms, correlated to parasympathetic activity) were calculated.

##### Frequency domain variables

Total power in the frequency range (0-0.40 Hz) was divided into: very low frequency (VLF: < 0.04 Hz), low frequency (LF: 0.04-0.15 Hz, modulated by SNS), and high frequency (HF: 0.15-0.4 Hz, modulated by parasympathetic nervous system (PNS)). LF and HF measured in normalized units, which represent the relative value of each power component in proportion to the total power minus the VLF component. The LF/HF ratio, regarded as an index of cardiac sympathetic/parasympathetic tone balance, was also calculated.

### Measurement of serum adipocytokines

Blood samples were taken after overnight fasting; serum was separated, stored at -80°C, and were analyzed at a later time. Serum leptin levels were measured using radioimmunoassay (Millipore, Billerica, USA). Serum adiponectin and TNF-α levels were measured using a commercially available enzyme-linked immunosorbent assay (ELISA, R&D Systems Inc., Minneapolis, USA).

An automated device (VP-1000; Colin, Japan) was used to measure arterial pulse wave velocity (PWV) and ankle-brachial index (ABI). The insulin resistance status was evaluated by the HOMA-IR index. The HOMA-IR was calculated by the formula: [fasting insulin (uIU/mL)x fasting blood glucose (mmol/L)]/22.5. The HOMA-IR score was available only in 128 patients not receiving exogeneous insulin.

### Statistical analysis

Statistical analysis was performed using SPSS 14.0 (SPSS Inc, Chicago, IL, U.S.A). Data are reported as mean standard deviation (SD) for variables which are normally distributed or as median (minimum-maximum) for variables which are not normally distributed or as number of participants (percentages). Non-normally distributed variables, that is, TG, hsCRP and HOMA-IR were transformed as natural logarithm before analysis. The categorical variables of the groups were compared by Chi-square test. The significance of the mean differences between patients with CAN and those without CAN was evaluated with Student's *t*-test. One-way ANOVA was used to evaluate differences of means among multiple groups. Correlation between plasma adipocytokines and other clinical parameters were analyzed by Pearson's or Spearman's correlation analysis. We used the odds ratio (OR) as a measure of the association between adipocytokines or other variables and presence of CAN in multivariate logistic regression analysis.

## Results

### The clinical characteristics of the participants

The general characteristics of the study participants are presented in Table 1. The age of the participants was 52.4 ± 10.0 years, and the mean duration of diabetes was 6.1 years. The mean body mass index (BMI) was 24.6 ± 3.7 kg/m^2^. Forty-five (31.7%) patients were treated for hypertension; 2 (1.3%) with angiotensin converting enzyme (ACE) inhibitors, 29 (19.3%) with angiotensin receptor blockers, 16 (10.7%) with calcium channel blockers.

**Table 1:** General characteristics of the participants

age	52.4 ± 10.0
Men/Women (%)	94/48 (66.2/33.8)
duration of DM(year)	6.1 ± 5.0
Body mass index (kg/m^2^)	24.6 ± 3.7
Systolic BP (mmHg)	127.8 ± 14.7
Diastolic BP (mmHg)	78.1 ± 8.6
HbA1_C _(%)	7.9 ± 1.9 (5.5-16.2)
FPG (mg/dL)	141 (73-377)
eGFR (mL/min/1.73 m^2^)	80.6 ± 16.0
Total cholesterol (mg/dL)	168.6 ± 32.0
LDL-cholesterol (mg/dL)	95.2 ± 27.7
Hypertension, n (%)	46 (32.1)
HDL-cholesterol (mg/dL)	47.7 ± 15.2
Triglycerides (mg/dL)	126 (38-1013)
hsCRP (mg/dL)	0.08 (0.03-11.42)
apolipoprotein B (mg/dL)	78.2 ± 22.3
ABI	1.11 ± 0.12
PWV (cm/sec)	1515 ± 248
HOMA-IR	2.69 (0.09-29.01)
smoking, n (%)	47(33.1)
Alcohol, n (%)	62 (43.7)
treatment modality no medication, n (%)	32 (22.7)
OHA, n (%)	97 (68.8)
OHA + Insulin, n (%)	5 (3.5)
Insulin, n (%)	7 (5.0)

Data are shown as mean ± SD, median (minimum-maximum) or as n (%).

FPG: fasting plasma glucose; eGFR: estimated glomerular filtration rate;

LDL: Low density lipoprotein; HDL: high density lipoprotein;

hsCRP: high-sensitivity C-reactive protein; ABI: ankle-brachial index;

PWV: pulse wave velocity; HOMA-IR: Homeostasis model assessment-insulin resistance;

OHA: oral hypoglycemic agent

### Prevalence of CAN

The abnormalities detected in the tests for CAN are shown in Table 2. Of the 142 patients, 46 (32.4%) were defined as having CAN. The incidence of CAN was more prevalent in women as compared to men (men: 27.7%, women: 41.7%). Of the five tests used to determine the CAN score, abnormal responses were most frequently found in the heart rate response to the deep breathing test. The postural blood pressure test yielded the fewest abnormal responses. Among the 21 patients with newly diagnosed diabetes at baseline, 3 (14.3%) patients were found to have CAN.

**Table 2:** Prevalence of CAN in all patients

	Men			women			total		
	
	Normal	Borderline	Abnormal	Normal	Borderline	Abnormal	Normal	Borderline	Abnormal
HR response to	51	19	24	16	11	21	67	30	45
deep breathing	(54.3)	(20.2)	(25.5)	(33.3)	(22.9)	(43.8)	(47.2)	(21.1)	(31.7)

lying-to-standing	65	13	16	31	9	8	96	22	24
HR response	(69.1)	(13.8)	(17)	(64.6)	(18.8)	(16.7)	(67.6)	(15.5)	(16.9)

valsalva	59	16	19	29	11	8	88	27	27
maneurver	(62.8)	(17)	(20.2)	(60.4)	(22.9)	(16.7)	(62)	(19)	(19)

Postural BP	76	18		32	15	1	108	33	1
change	(80.9)	(19.1)		(66.7)	(31.3)	(2.1)	(76.1)	(23.2)	(0.7)

Sustained	57	24	13	25	10	13	82	34	26
handgrip test	(60.6)	(25.5)	(13.8)	(52.1)	(20.8)	(27.1)	(57.7)	(23.9)	(18.3)

CAN(-/+)	68(72.3)/26(27.7)			28(58.3)/20(41.7)			96(67.6)/46(32.4)		

Data are expressed as n (%). HR, heart rate; CAN, cardiac autonomic neuropathy

### The comparison of mean adipocytokine levels and clinical variables including HRV parameters according to the presence of CAN

The clinical characteristics and laboratory findings according to the presence of CAN are presented in Table 3. The mean levels of leptin, TNF-α and adiponectin were not significantly different between the groups with and without CAN, although the adiponectin level tended to be higher in the CAN group (3138 vs 4185 ng/ml, p = 0.072). Compared to the patients without CAN, patients with CAN were older and had a longer duration of diabetes. The patients with CAN were more likely to have hypertension than those without CAN (p = 0.01). The mean levels of PWV were borderline significantly higher in patients with CAN compared to patients without CAN (p = 0.05). Notably, the patients with CAN had significantly higher levels of HOMA-IR (p = 0.016) compared with patients without CAN. However, there were no significant differences in HbA1_C _levels and lipid profiles between the two groups.

**Table 3:** The clinical characteristics and laboratory findings of patients according to the presence of CAN

	CAN-	CAN+	P-value
Age	51.0 ± 9.7	55.2 ± 10.2	0.021
Men/Women (%)	68/28(71/29)	26/20(56.5/43.5)	0.048
Duration of DM(year)	5.5 ± 4.7	7.5 ± 5.3	0.025
Leptin (ng/ml)	7.25 ± 7.41	8.14 ± 7.31	0.506
TNF-α (pg/ml)	1.93 ± 1.13	2.95 ± 5.51	0.220
Adiponectin (ng/ml)	3138 ± 3010	4185 ± 3615	0.072
Body mass index (kg/m^2^)	24.4 ± 3.6	25.0 ± 3.8	0.435
Systolic BP (mmHg)	126.3 ± 14.2	130.9 ± 15.4	0.080
Diastolic BP (mmHg)	77.6 ± 8.2	79.2 ± 9.3	0.308
HbA1_C _(%)	8.0 ± 2.1	7.9 ± 1.7	0.756
FPG (mg/dL)*	139 (73-377)	148 (88-340)	0.324
eGFR (mL/min/1.73m2)	81.2 ± 14.6	79.3 ± 19.0	0.531
Total cholesterol (mg/dL)	169.1 ± 31.9	167.4 ± 32.6	0.775
LDL-cholesterol (mg/dL)	96.5 ± 28.0	92.6 ± 27.2	0.438
Hypertension, n (%)	24 (25.3)	21 (46.7)	0.010
HDL-cholesterol (mg/dL)	47.6 ± 16.1	47.8 ± 13.2	0.943
Triglycerides (mg/dL)*	137 (38-1013)	121 (52-356)	0.118
hsCRP*	0.09 (0.03-6.59)	0.08 (0.03-11.42)	0.780
Apolipoprotein B (mg/dL)	79.3 ± 23.6	75.7 ± 19.3	0.462
ABI	1.11 ± 0.14	1.12 ± 0.06	0.473
PWV (cm/sec)	1488 ± 242	1575 ± 252	0.050
HOMA-IR*	2.58 (0.09-15.82)	2.87 (0.55-29.01)	0.016
Smoking (%)	32 (33.3)	15 (32.6)	0.545
Alcohol (%)	38 (39.6)	24 (52.2)	0.088
Treatment modality			0.131
No medication	27 (28.1)	5(11.1)	
OHA (n/%)	63 (65.6)	34 (75.6)	
OHA + Insulin (n/%)	3 (3.1)	2 (4.4)	
Insulin (n/%)	3 (3.1)	4 (8.9)	

Data are shown as mean ± SD, or as n (%). BMI: Body mass index; FPG: fasting plasma glucose; HbA1_C_: hemoglobin A1_C_; eGFR: estimated glomerular filtration rate; LDL: Low density lipoprotein; HDL: high density lipoprotein; OHA: oral hypoglycemic agent *Natural logarithmic transformations were performed before analysis

Table 4 shows the comparisons of parameters of time and frequency domain of HRV according to the presence or absence of CAN. The SDNN in the upright position was significantly decreased in patients with CAN compared to patients without CAN (p = 0.003).

**Table 4:** Heart rate variability parameters by time and frequency domain of patients according to the presence of CAN

Heart rate variability	CAN-	CAN+	P-value
Supine			
HR	72.8 ± 11.3	76.0 ± 11.4	0.114
SDNN (ms)	28.4 ± 14.2	23.9 ± 14.8	0.081
RMSSD (ms)	18.9 ± 15.7	16.8 ± 17.1	0.459
LF (nu)	63.2 ± 20.5	61.9 ± 22.9	0.742
HF (nu)	36.8 ± 20.5	38.1 ± 22.9	0.742
LF/HF	3.4 ± 4.5	3.5 ± 4.9	0.865
Upright			
HR	83.5 ± 29.6	81.4 ± 18.4	0.665
SDNN (ms)	25.2 ± 13.8	18.3 ± 10.3	0.003
RMSSD (ms)	19.6 ± 18.3	14.5 ± 10.6	0.081
LF (nu)	65.0 ± 20.1	59.4 ± 21.4	0.130
HF (nu)	35.9 ± 21.0	39.5 ± 21.8	0.346
LF/HF	3.9 ± 5.7	3.3 ± 4.8	0.466

Data are shown as means ± SD. HR: Heart rate; SDNN: standard deviation of normal-to-normal RR intervals; RMSSD: square root of the average of the sum of the squares of the differences between adjacent NN intervals; LF: Low frequency; HF: High frequency

The participants were divided into four groups according to the scores of CAN as previously described. The levels of leptin and adiponectin demonstrate an increasing trend as the score of CAN increased (p = 0.05 and p = 0.036, respectively) (Table 5).

**Table 5:** The comparisons of mean levels of adipocytokines according to the scores of CAN

Score of CAN	Leptin (ng/ml)	TNF-α(pg/ml)	adiponectin(ng/ml)
0	5.44 ± 4.4	1.64 ± 0.32	2718 ± 2759
1	7.38 ± 7.5	2.00 ± 1.26	3229 ± 3096
2	8.08 ± 7.3	3.11 ± 5.82	4003 ± 3307
3	12.55 ± 12.3	1.70 ± 0.49	5434 ± 5293
p for trend	0.05	0.784	0.036

Data are shown as mean ± SD.

### Bivariate correlation between leptin, TNF-α, adiponectin and clinical parameters

Bivariate correlation analyses between leptin, TNF-α, adiponectin and various clinical parameters are shown in Table 6. Regarding serum leptin levels, significant positive correlation was found with body mass index (BMI), triglyceride (TG), high-sensitivity C-reactive protein (hsCRP) and HOMA-IR. Serum leptin levels exhibited a borderline significant positive correlation with CAN score. Moreover, the positive correlation between serum leptin with BMI, TC and TG remained significant after adjustment for age and sex (r = 0.41, p < 0.001; r = 0.29, p = 0.027; r = 0.53, p < 0.001, respectively).

**Table 6:** Correlation of serum adiponectin, leptin, and TNF-α with clinical variables

	Leptin		TNF-α		adiponectin	
	r	p	r	p	R	p
Age	0.119	0.159	0.222	0.008	0.129	0.126
Duration of DM	-0.057	0.500	0.078	0.359	0.189	0.024
Body mass index	0.481	< 0.001	0.049	0.562	-0.229	0.006
Systolic BP	0.138	0.103	0.151	0.073	-0.172	0.041
Diastolic BP	-0.004	0.965	0.065	0.442	-0.210	0.012
HbA1_C_	-0.139	0.103	0.019	0.827	0.037	0.666
FPG*	-0.077	0.367	-0.083	0.331	0.016	0.851
eGFR	-0.06	0.491	-0.151	0.080	-0.138	0.111
Total cholesterol	0.075	0.392	0.068	0.435	-0.158	0.070
LDL-cholesterol	-0.030	0.726	0.060	0.483	-0.096	0.262
HDL-cholesterol	-0.033	0.708	0.108	0.218	0.053	0.548
Triglycerides*	0.254	0.003	-0.710	0.405	-0.244	0.004
HsCRP*	0.264	0.005	0.304	0.001	-0.321	0.001
Apolipoprotein B	0.049	0.638	0.007	0.950	-0.344	0.001
mean ABI	0.008	0.929	0.034	0.699	0.077	0.374
mean PWV	0.031	0.722	0.201	0.020	-0.069	0.943
HOMA-IR*	0.301	0.001	-0.051	0.595	-0.174	0.071
CAN score	0.160	0.057	0.117	0.164	0.177	0.035
Leptin	-	-	-0.087	0.304	-0.048	0.570
TNF-α	-0.087	0.304	-	-	0.028	0.736
Adiponectin	-0.048	0.570	0.028	0.736	-	-

FPG: fasting plasma glucose; eGFR: estimated glomerular filtration rate; LDL: Low density lipoprotein; HDL: high density lipoprotein; hsCRP: high sensitivity c-reactive protein; ABI: ankle-brachial index; PWV: pulse wave velocity; HOMA-IR: homeostasis model assessment-insulin resistance

*Natural logarithmic transformations were performed before analysis

Serum TNF-α levels showed a positive correlation with age, hsCRP and mean PWV. After adjustment for age and sex, the serum TNF-α level consistently showed positive correlation with the SBP and mean PWV (r = 0.26, p = 0.05; r = 0.29, p = 0.025).

Serum adiponectin levels showed a significant negative correlations with BMI, systolic blood pressure (SBP), diastolic blood pressure (DBP), TG, hsCRP and apolipoprotein B. In addition, serum adiponectin levels demonstrated a significant positive correlations with duration of DM and CAN score. After adjustments for age and sex, the adiponectin level showed a negative correlation with the BMI, SBP, DBP, TG, apoB and hsCRP (r = -0.38, p = 0.003; r = -0.32, p = 0.013; r = -0.28, p = 0.034; r = -0.26, p = 0.043; r = -0.32, p = 0.014; r = -0.26, p = 0.047) (Adjusted correlation data not shown).

### Bivariate correlation of serum leptin, TNF-α, and adiponectin with HRV parameters

Correlation of serum leptin, TNF-α, and adiponectin levels with HRV parameters are shown in Table 7. The serum leptin levels exhibited a negative correlation with LF in the upright position (r = -0.2, p = 0.037). Regarding TNF-α, a significant negative correlation was observed with SDNN and RMSSD in the upright position (r = -0.19, p = 0.023; r = -0.2, p = 0.019). Adiponectin was not related to any HRV variables.

**Table 7:** Correlation between parameters of HRV and serum adipocytokine levels

	leptin		TNF-α		adiponectin	
	r	p	r	p	r	p
SDNNsupine	0.10	0.244	-0.03	0.692	-0.05	0.541
SDNNupright	0.09	0.311	-0.19	0.023	-0.06	0.493
RMSSDsupine	0.03	0.771	-0.12	0.159	-0.03	0.715
RMSSDupright	0.14	0.109	-0.20	0.019	-0.04	0.599
LFsupine	0.03	0.680	0.14	0.107	-0.09	0.275
LFupright	-0.18	0.037	0.08	0.327	-0.07	0.425
HFsupine	-0.04	0.683	-0.14	0.107	0.09	0.277
HFupright	0.15	0.072	-0.07	0.418	0.04	0.605
LFHFsupine	0.02	0.815	0.13	0.133	-0.12	0.171
LFHFupright	-0.15	0.086	0.10	0.257	-0.09	0.286

SDNN: Standard deviation of normal-to-normal RR intervals; RMSSD: square root of the average of the sum of the squares of the differences between adjacent NN intervals; LF: Low frequency; HF: High frequency Spearman's correlation analysis was used for the statistical analyses

SDNN in supine showed a negative correlation with age, duration of DM, SBP, DBP (r = -0.24, p = 0.004; r = -0.22, p = 0.09; r = -0.20, p = 0.017; r = -0.26, p = 0.002, respectively) and a positive correlation with HDL-C (r = 0.28, p = 0.001). The LF/HF ratio in supine showed a positive correlation with BMI, DBP and HOMA-IR (r = 0.30, p = 0.019; r = 0.26, p = 0.047; r = 0.27, p = 0.005) (Data not shown).

### Multiple logistic regression analysis for the relationship of adipocytokines with presence of CAN

To examine the relationship of serum adipocytokines with the presence of CAN, a multivariate logistic regression analysis was performed (Table 8).

**Table 8:** Multivariate logistic regression analysis with presence or absence of CAN as the dependent variable

Independent variable	Odds ratio (95% CI)	P-value (p for trend*)
Age	1.04 (0.95-1.14)	0.453
duration of diabetes	1.25 (1.07-1.47)	0.006
adiponectin		0.005 (0.022*)
1st tertile	1	
2nd tertile	14.1 (1.9-105.6)	0.01
3 rd tertile	51.6 (4.8-450)	0.001
HTN	2.19 (0.51-9.34)	0.291
retinopathy	0.75 (0.08-6.86)	0.796
mean PWV	1.003 (0.99-1.01)	0.051
HOMA-IR	5.47 (1.80-16.57)	0.003
Smoking	1.23 (0.311-4.89)	0.765
Alcohol	2.02 (0.55-7.45)	0.289

HTN: Hypertension; PWV: pulse wave velocity;

HOMA-IR: homeostasis model of assessment-insulin resistance

In multivariate analysis, only the duration of diabetes, HOMA-IR, and adiponectin were significantly associated with the presence of CAN. An increased duration of diabetes and higher HOMA-IR independently increased the odds for the presence of CAN (OR = 1.25, [1.07-1.47]; OR = 5.47, [1.80-16.57], respectively). The relative risks for the presence of CAN were 14.1 and 51.6 for the adiponectin 2^nd^, 3^rd ^tertiles when compared with first tertile (p-value for trend = 0.022).

## Discussion

In the present study, the higher serum adiponectin levels were associated with an increased probability for the presence of CAN. In addition, the measured score of CAN correlated with the serum adiponectin levels.

Among the adipocytokines, adiponectin is widely known as a beneficial hormone to diabetes and CVD due to its anti-inflammatory, anti-atherogenic and anti-diabetic properties [20]. Park et al. reported that central adiponectin increased pancreatic beta-cell mass and attenuated insulin resistance in diabetic rats [21]. In our study, the multiple links of adiponectin to several metabolic risk factors like BMI, BP, TG, apoB, and hsCRP are all coherently in line with the hypothesis that adiponectin is a protective factor [22]. However, increased adiponectin levels were associated with a higher probability of CAN which is independent of the traditional and nontraditional risk factors in mutivariate logistic regression analysis in this study. Also, with an increasing score of CAN, the levels of adiponectin showed a tendency to increase. It is not clear why serum adiponectin level is positively correlated with the presence and severity of CAN. One of the possible explanations is that in response to endothelial dysfunction, oxidative stress, insulin resistance promoting autonomic dysfunction, the serum adiponectin may be compensatorily increased. Conversely, another possible explanation is that adiponectin may worsen diabetic CAN, although this appears to be contrary to the concept of adiponectin as a beneficial hormone.

In the present study, we included the evaluation of the relationship of HRV parameters with the serum adipocytokine levels. SDNN is the most representative parameter of HRV. Therefore, low SDNN represents a low HRV, which primarily indicates a reduction in dynamic complexity [5]. DM is associated with lowered SDNN [23]. In this study, the SDNN in supine showed a negative correlation with age, BP and a positive correlation with HDL-C. RMSSD is specific for the parasympathetic modulation. Decrease in RMSSD accompanying lowered SDNN is related to high risk of cardiac disease development [24].

Our data demonstrated a negative correlation between the TNF-α and SDNN and RMSSD. These results suggest that the TNF-α promoting inflammatory reaction may be associated with low HRV and PNS dysfunction in T2DM patients.

Spectral analysis of HRV (frequency domain measures) is another tool to evaluate CAN. The LF band reflects SNS and the HF band reflects PNS activity [25]. Reduced PNS activity has been found in a number of cardiac pathologies including diabetic cardiomyopathies. The reduced PNS activity is also believed to account for much of the reduced HRV. The LF/HF ratio indicates overall balance of the ANS. The higher LF/HF ratio reflects the domination of SNS while a lower one reflects domination of the PNS. In our data, the LF/HF ratio in supine showed positive correlation with insulin resistance assessed by HOMA-IR. This result is in line with studies showed a pathogenic link between autonomic dysfunction and insulin resistance [26,27].

Serum leptin levels showed a negative correlation with the LF. This indicated that leptin showed negative correlation with parameters of SNS activity and autonomic balance. These results are inconsistent with those of previous studies, which generally suggest that leptin is associated with sympathetic activation [11]. However, the evidence relating leptin to SNS in type 2 diabetes is less clear. The majority of cross-sectional clinical studies reporting a positive correlations between circulating leptin levels and SNS activation indexed by HRV have been performed in both lean and obese non-diabetic individuals and not in T2DM patients. Studies regarding the relationship between leptin and HRV in type 2 diabetes are rare and have been inconclusive.

In agreement with a previous report by Piestrzeniewicz et al., in our present study, adiponectin was not related to any HRV parameters [28]. Information collected from other studies conducted in groups of patients with T2DM, suggests that there are possible links between hypoadiponectinemia and SNS [29,30]. Wakabayashi et al. showed an independent negative association between the serum adiponectin concentration and 24 hour LF/HF ratio [16]. Boer-Martins et al. reported HRV correlated positively with serum adiponectin [31]. However, there is still limited information on the relationship between plasma adiponectin, T2DM and cardiac autonomic nervous function.

Although cardiac autonomic function testing utilizing the heart rate variability by Ewing's method is sensitive, noninvasive and reproducible, these time domain and frequency domain methods are less affected by the examiner's skill and cooperation of the patients. The strengths of our study are that we examined both Ewing's method and time and frequency domain parameters for the evaluation of HRV.

The prevalence of CAN in this cohort of Korean type 2 diabetic patients was 32.4%. Of these, 53.5% were diagnosed as having early CAN. This result is consistent with other studies [32,33]. In the study of Vinik et al., the prevalence of CAN was approximately 34% among individuals with type 2 diabetes, when diagnostic criteria are based upon at least two abnormalities in the autonomic function tests [27]. This is comparable with another Korean data by Moon et al., who reported that the prevalence of CAN in diabetics is 26.3% and 65.3% had early CAN [33]. Among the 21 patients with newly diagnosed diabetes at baseline, three (14.3%) showed CAN in our study.

In this study, we observed that the prevalence of CAN was higher in women than in men. In general, sex differences have been found in the onset of CAN, with men presenting with autonomic impairment earlier and more severity than women [34]. However, in our study, women participants were older than men (54.5 vs 51.3 years, p = 0.043). We think that this age factor may contribute to the prevalence of CAN. We did observe that patients with CAN were significantly older than patients without CAN, and that they also had significantly longer duration of DM, a higher prevalence of HTN, a higher levels of HOMA-IR and mean PWV. These observations are consistent with those of previous studies [35,36]. Especially, higher HOMA-IR levels were associated with the increased probability of CAN in multivariate logistic regression analysis. These findings emphasize the role of insulin resistance, not only in the etiology of the metabolic syndrome, but also as a determinant of cardiovascular autonomic regulation.

In accordance with the results of study by Moon et al., out of the five tests used to determine the CAN score, abnormal responses were most frequently found for the heart rate response to the deep breathing test [33]. In contrast, Ko et al., reported that the abnormal response to valsalva maneuver was the most frequent [37].

Since the hypoglycemia itself also influence the results of CAN, we reviewed medical records of all participants for find hypoglycemic attacks. Five patients experienced hypoglycemia during the last month previous to CAN tests. However, hypoglycemic events were infrequently developed (< 2 episodes). In addition, all patients experiencing hypoglycemia were classified as group of absence of CAN. Therefore, we think that the possibility of affecting the results of CAN of hypoglycemic attack in this study is remote.

Several limitations of our study should be addressed. First, due to the cross-sectional design, we cannot determine the causative relationship between adiponectin and CAN complications. Prospective studies are required to address this important question. Second, because our study population included individuals who received the autonomic function test, some characteristics of the present study population may be substantially different from other populations that did not perform complication study. Therefore, the generalizability of our study may be limited. Third, the present study included a small numbers of subjects. A larger number of patients should be analyzed for the confirmation of our results. However, our study is meaningful in that this is the first study in Korean T2DM patients for the evaluation of several adipocytokines and CAN. Moreover, to our knowledge, none of the previously published studies investigated the relationship of adipocytokines with HRV parameters in Korean T2DM.

In conclusion, the higher serum adiponectin levels were associated with an increased risk for the presence of CAN. Longer duration of diabetes and higher insulin resistance were independently associated with the presence of CAN in Korean T2DM patients. Serum adipocytokines such as leptin and TNF-α were significantly correlated with parameters of HRV, representative markers of CAN. Future prospective studies with larger numbers of patients are required to establish a direct relationship between plasma adipocytokine concentrations and the development or severity of CAN.

## Abbreviations

CAN: cardiac autonomic neuropathy; T2DM: type 2 diabetes mellitus; TNF- α: tumor necrosis factor- α; HRV: heart rate variability; LF: low frequency; HF: high frequency; SDNN: standard deviation of normal-to-normal RR intervals; RMSSD: square root of the mean of the squares of differences between successive RR intervals; HOMA-IR: homeostasis model assessment-insulin resistance; CVD: cardiovascular disease; SNS: sympathetic nervous system; AFT: autonomic function test; PNS: parasympathetic nervous system; PWV: pulse wave velocity; ABI: ankle-brachial index; ACE: angiotensin converting enzyme; BMI: body mass index; SBP: systolic blood pressure; FPG: fasting plasma glucose; TG: triglyceride; hsCRP: high sensitivity c-reactive protein; HDL-C: high density lipoprotein cholesterol; apoB: apolipoprotein B; eGFR: estimated glomerular filtration rate; OHA: oral hypoglycemic agent

## Competing interests

The authors declare that they have no competing interests.

## Authors' contributions

CHJ, CHK, SKK and JOM contributed to the design, analysis and interpretation of this study. BYK contributed to the collection of clinical and laboratory data. SHJ contributed to the writing of this manuscript. All authors read and approved the final manuscript.
